# Acceptability, feasibility, and impact of a pilot tuberculosis literacy and treatment counselling intervention: a mixed methods study

**DOI:** 10.1186/s12879-021-06136-1

**Published:** 2021-05-18

**Authors:** Stephanie Law, Boitumelo Seepamore, Olivia Oxlade, Nondumiso Sikhakhane, Halima Dawood, Sheldon Chetty, Nesri Padayatchi, Dick Menzies, Amrita Daftary

**Affiliations:** 1 Department of Global Health and Social Medicine, Harvard Medical School, 641 Huntington Avenue, Boston, MA 02115 USA; 2 McGill International TB Centre, Research Institute of the McGill University Health Centre, 1001 rue Decarie, Montreal, Quebec H4A 3J1 Canada; 3 School of Applied Human Sciences, University of KwaZulu-Natal, Howard College Campus, 238 Mazisi Kunene Rd, Glenwood, Durban, 4041 South Africa; 4 CAPRISA- MRC HIV-TB Treatment and Pathogenesis Unit, Doris Duke Medical Research Institute, Nelson R Mandela School of Medicine, University of KwaZulu-Natal, Private Bag X7, Congella, 4013 South Africa; 5 Department Medicine, Infectious Disease Unit, Greys Hospital, The Msunduzi, Town Hill, Pietermaritzburg, 3201 South Africa; 6 East Boom Community Healthcare Centre, 541 Boom Street, Pietermaritzburg, 3201 South Africa; 7 Department of Epidemiology, Biostatistics and Occupational Health, McGill University, 1020 Pine Ave W, Montreal, Quebec, H3A 1A2 Canada; 8 School of Global Health and Dahdaleh Institute of Global Health Research, York University, 88 The Pond Rd, North York, ON M3J 2S5 Canada

**Keywords:** Tuberculosis, Mixed methods intervention, Provider engagement, Education and counselling, South Africa

## Abstract

**Background:**

There is a need for innovative strategies to improve TB testing uptake and patient retention along the continuum of TB care early-on in treatment without burdening under-resourced health systems. We used a mixed methods approach to develop and pilot test a tuberculosis literacy and counselling intervention at an urban clinic in KwaZulu Natal, South Africa, to improve TB testing uptake and retention in tuberculosis care.

**Methods:**

We engaged in discussions with clinic staff to plan and develop the intervention, which was delivered by senior social work students who received one-week training. The intervention included: 1) group health talks with all patients attending the primary clinic; and 2) individual counselling sessions, using motivational interviewing techniques, with newly diagnosed tuberculosis patients. We compared social work students’ tuberculosis knowledge, attitudes, and practices before and after their training. We assessed the change in number of tuberculosis diagnostic tests performed after implementation via an interrupted time series analysis with a quasi-Poisson regression model. We compared pre- and post-intervention probabilities of treatment initiation and completion using regression analyses, adjusting for potential baseline confounders. We conducted focus groups with the students, as well as brief surveys and one-on-one interviews with patients, to assess acceptability, feasibility, and implementation.

**Results:**

During the study period, 1226 individuals received tuberculosis diagnostic testing and 163 patients started tuberculosis treatment, of whom 84 (51.5%) received individual counselling. The number of diagnostic tuberculosis tests performed increased by 1.36 (95%CI 1.23–1.58) times post-intervention, adjusting for background calendar trend. Probabilities of TB treatment initiation and treatment completion increased by 10.1% (95%CI 1.5–21.3%) and 4.4% (95%CI -7.3-16.0%), respectively. Patients found the counselling sessions alleviated anxiety and increased treatment self-efficacy. Social work students felt the clinic staff were collaborative and highly supportive of the intervention, and that it improved patient engagement and adherence.

**Conclusions:**

Engaging clinic staff in the development of an intervention ensures buy-in and collaboration. Education and counselling before and early-on in tuberculosis treatment can increase tuberculosis testing and treatment uptake. Training junior social workers can enable task-shifting in under-resourced settings, while addressing important service gaps in tuberculosis care.

**Supplementary Information:**

The online version contains supplementary material available at 10.1186/s12879-021-06136-1.

## Background

South Africa has the world’s eighth highest incidence rate of tuberculosis (TB) [1]. Each year, there are 300,000 people who develop TB and about 63,000 TB deaths, of which about two-thirds are in people living with HIV (PLWH) [2]. Engaging people in the health care system to get tested, and then retaining them through the whole continuum of TB care, that is, keeping them engaged in the health system from the time of their initial TB test through to treatment completion, are major challenges for the national TB program. Nationally, about 18% of people estimated to have active TB are not diagnosed, and an estimated 15% of those diagnosed do not start TB treatment. Of those who start, approximately 24% do not complete the standard six-month treatment course [3].

Closely related to the problem of patient engagement and retention is medication adherence, which refers to whether patients take all their treatment as prescribed. The main global approach to promote TB treatment adherence and retention is directly observed therapy (DOT), wherein a designated supervisor such as a health provider or community member observes patients ingest their medicines. However, this approach imposes a heavy demand on already fragile health resources and is thus inconsistently implemented in many parts of South Africa; patients are formally observed only once per week or month, if at all, with little to no support during the interim periods. Other reported limitations of DOT include its failure to address pre-treatment losses to follow-up and its lack of meaningful patient engagement [4–6]. DOT also ignores some important barriers to adherence and retention-in-care, including poor treatment literacy, conflicting attitudes and beliefs about TB Treatment, lack of social support, and low motivation and other related characteristics [7]. Innovative strategies that engage patients early on to prevent pre-treatment losses to follow-up, as well as during TB care to resolve unaddressed barriers to adherence, without burdening under-resourced health systems, are urgently needed [8, 9].

Patient education and counselling, deemed crucial to supporting patients on long-term treatment, is a pillar of HIV care and widely standardized in South Africa [10, 11]. It can fill in important treatment knowledge gaps, address acute or long-term barriers to adherence, nurture the provider-patient relationship, and provide necessary social or emotional support. However, although it is recommended by the National TB Programme [10] and globally under WHO’s guidelines for TB treatment and care [12], their provision to people with TB varies greatly across the country. Higher burden provinces such as KwaZulu-Natal (KZN) face especially dire operational constraints and provide limited, if any, treatment education or counselling to TB patients. To fill this service gap, we pilot tested an intervention that trained junior social work interns to provide TB education and counselling at an urban primary care clinic in KZN, with the goal of task-shifting from clinic staff while improving TB testing uptake and patient retention-in-care.

## Methods

Our study followed a mixed-methods intervention design [13] to develop, implement and evaluate a pilot TB treatment literacy and counselling intervention. Qualitative data was collected and analyzed before the intervention to support its development, as well as during and after the intervention to explain quantitative outcomes. We followed the STROBE Statement [14] and the TIDieR checklist [15] for reporting observational and intervention studies, respectively.

### Setting

This study was conducted at an urban primary health care clinic in Pietermaritzburg, capital of KZN, the province with the second highest TB incidence rate in the country (685 cases per 100,000 persons) [16]. All patients attending the clinic are screened for TB using a six-item risk assessment. Sputum is collected from those who screen positive and tested using GeneXpert MTB/RIF. Under routine care at the clinic, a registered nurse practitioner initiates and monitors treatment for all TB patients without documented resistance to rifampicin; although recommended, DOT is not practiced for most patients. All patients are first given 1 week of medication to take home and are followed up after the first week. Thereafter, patients are given their medications monthly to be taken with or without an appointed treatment supervisor in the community, at home, or at work. Those who experience any major adverse effects or adherence issues are followed up more regularly, on a case-by-case basis. Complex cases are referred to the on-site physician and rifampicin-resistant TB patients are referred to a tertiary facility. The TB nurse offers a limited degree of information about TB to new patients; those who are co-infected with HIV receive standard HIV counselling at the start of antiretroviral therapy (ART) and thereafter as needed, and see an HIV nurse for ART monitoring. Only patients experiencing severe difficulties are referred to a social worker. The clinic regularly hosts 3 to 5 social work students in their final year of studies for a six-month academic practicum to build skills, supervised by the site social worker and clinic manager.

### TB literacy and treatment counselling intervention

We developed an intervention framework that drew on components from other published TB counselling programs (e.g. [17–21]) and from the strengths-based approach, a social work practice theory that focuses on individuals’ strengths and resources in order to create opportunities and achieve their goals [22]; it is a client-centred, empowerment-based practice that promotes self-competence rather than the stigmatising notion of deficit. The framework was refined via a focus group discussion with clinic staff (Table 1 for details). The final intervention (Additional file 1: Box 1) comprised: 1) group health talks to all patients attending the clinic, which explained TB symptoms, testing and treatment, to motivate TB testing and treatment; and 2) two one-on-one TB counselling sessions for patients newly diagnosed with TB (the first, within the first week of starting treatment and the second, after 4 to 8 weeks), using motivational interviewing techniques to develop a personalized adherence plan (see Additional file 1: Box 2 for a copy of the patient adherence plan that guided discussions and was kept by each patient). The principles of motivational interviewing are closely aligned with the strengths-based approach and include: collaboration with the patient and acknowledging their expertise on their health; evoking a readiness to change and setting flexibly changing goals; and respecting a patient’s autonomy and empowering them to make changes on their own accord [23, 24]. Interventions based on motivational interviewing have been successful in improving patient retention in other clinical settings, such as for individuals seeking treatment for substance abuse [25] and people living with HIV on antiretroviral therapy [26].

**Table 1:** Description of quantitative and qualitative methods

Purpose	Description of methods
Refine intervention framework and counseling training	Quantitative**:** None Qualitative**:** We invited clinic staff involved in TB/HIV care to a focus group discussion (FGD) to discuss the barriers to retention in TB care and to obtain their feedback on the intervention. The FGD was facilitated by a trained study team member (BS) in English and isiZulu and followed a semi-structured, open-ended interview guide. We thematically analyzed transcripts and applied the findings accordingly.
Assess the impact of counsellor training	Quantitative**:** Knowledge, attitudes and practices (KAP) surveys (Additional file 1: Box 3) were administered to TB counsellors pre- and post-training. We compared pre-and post-training responses to the KAP survey knowledge questions (20 questions) using Fisher’s exact test and to the attitude questions (21 questions) using Wilcoxon rank-sum test (α < 0.10). We compared total knowledge scores pre- and post-training using a Wilcoxon rank-sum test. Qualitative**:** A trained social worker conducted a FGD with TB counsellors at the end of the intervention (“post-intervention FGD”), following a semi-structured interview guide.
Assess implementation and refine intervention	Quantitative: None Qualitative: TB counsellors recorded notes and memos after health talks and counselling sessions, which were reviewed by the study coordinator (NS) and a study team member (BS), discussed with the study team, and informed any ongoing changes to the intervention, as needed.
Assess impact on TB testing	Quantitative: We performed an interrupted time series analysis using a quasi-Poisson regression model, including calendar month as a fixed effect to account for the background seasonal trend, to compare the weekly number of TB tests pre- and post-intervention. Qualitative: Post-intervention FGD
Assess impact on TB treatment initiation	Quantitative**:** We performed univariate and multivariate (adjusting for age, sex and calendar month, without imputing any missing data on confounders) binomial regressions to compare probabilities of treatment initiation in the two periods, and a Mann-Whitney non-parametric test to compare the median treatment delay (i.e., the number of days from testing to starting TB treatment). Qualitative**:** Post-intervention FGD
Assess impact on TB treatment outcomes	Quantitative: We performed univariate and multivariate logistic regression analyses (adjusting for age, sex, smear status, and HIV and ART status, without imputing missing confounders) to compare the probability of TB treatment completion. In the main analysis, we compared the study and historical control periods and included all new TB patients in the study period regardless of whether they enrolled into the study (i.e. an intention-to-treat analysis). In our sensitivity analyses, we compared: patients enrolled in the study period to all other patients (in both the study and historical control period); patients enrolled in the study to patients in the historical control period; and patients enrolled to patients not enrolled in the study during study period (Additional file 1: Table 8). Qualitative: Post-intervention FGD
Explore counsellors’ and patients’ perspectives on the impact, acceptability and feasibility of the intervention	Quantitative**:** Descriptive analyses of brief exit surveys (Additional file 1: Box 4) that were administered to all enrolled patients who received both counselling sessions. Qualitative**:** In addition to the post-intervention FGD and brief patient exit surveys, a purposive sample of enrolled patients (aiming for maximum variation in patient characteristics based on age, gender, education, and TB/HIV history) were recruited for one-on-one, in-depth interviews with the study coordinator (NS), following a semi-structured interview guide. All of the collected data (survey responses and transcripts) were thematically analyzed using a constant comparative approach.

*Abbreviations*: *FGD* Focus group discussion, *KAP* Knowledge, attitudes and practices

### TB counsellor training

Social work students in their final year of studies and completing their practicums at the clinic between May and September 2018 were invited to participate as TB counsellors. Consenting students (hereon referred to as “TB counsellors”) were trained for 1 week on counselling skills; TB symptoms, diagnosis, and treatment; and motivational interviewing techniques (details in Additional file 1: Table 1). Their execution of study activities was monitored by the site social worker, clinic manager and study coordinator, who reviewed counselling logs to give weekly feedback and mentorship in consultation with social work experts on our team.

### Patient recruitment and sampling

All newly diagnosed TB patients (without documented resistance to rifampicin) initiating treatment at the clinic between May 21 and Sep 4, 2018 inclusive (hereon referred to as the “study period”) were eligible to receive individual counselling through the study. TB staff were reminded to refer all TB patients starting treatment to the study coordinator and recruitment signs were posted in the TB rooms. After referral, the study coordinator met privately with eligible patients (or their caregiver if the patient was under 18 years old) to explain the study and obtain written informed consent. Enrolled patients (or caregivers) received counselling sessions in a private area on-site, in addition to standard of care. All enrolled patients who attended both counselling sessions were given a brief exit survey to complete after the second session to gather basic feedback on intervention acceptability. A subsample of these patients was recruited for in-depth interviews using a qualitative purposive sampling framework of maximum variation (based on gender, age and previous history of TB) to gather more insights.

### Data collection and analysis

#### Overview

Quantitative and qualitative data were collected in parallel throughout the study, analyzed separately, then integrated to corroborate findings. Methods of data collection and analysis are described ahead and in Table 1. All statistical analyses of quantitative data were performed using R (v3.5.1).

#### TB diagnostic testing

We recorded the number of TB diagnostic tests performed and results (GeneXpert, culture testing and chest x-rays) during the study period from the clinic test register. For comparison, we collected the same TB testing data from the 12-month period preceding the intervention (May 1, 2017 to May 20, 2018 inclusive). We performed an interrupted time series analysis using a quasi-Poisson regression model to compare the weekly number of TB tests observed post-intervention to the number that was expected, based on the 12 months preceding the intervention.

#### Treatment initiation

We collected sociodemographic data, TB testing dates and results and, if applicable, treatment start date from the TB register for patients diagnosed with bacteriologically confirmed TB (i.e. culture or GeneXpert positive) during the study period, and for comparison, during the same calendar period in the previous year (May 22 to Sep 5, 2017; hereon referred to as the “historical control period”). Treatment initiation dates were included if they were recorded before the end of September for each time period, allowing up to a 3-week treatment delay for those diagnosed at the start of September. We performed descriptive, univariate, and multivariate analyses to compare the probability of treatment initiation, as well as treatment delays (defined as the number of days from testing to starting TB treatment) (Table 1).

#### Treatment outcomes

We extracted TB/HIV diagnosis and final 6-month TB treatment outcomes data on patients who started TB treatment during the study and historical control periods. Treatment outcomes (cure; complete; fail death; moved/transferred; or lost to follow-up) were defined according to WHO guidance [27]. TB counsellors collected additional sociodemographic data from enrolled patients via a standardized questionnaire. We performed univariate and multivariate logistic regression analyses to compare the probabilities of completing treatment; additional sensitivity analyses comparing different groups were conducted (Table 1).

#### Acceptability and feasibility

We assessed intervention acceptability, feasibility and implementation via the brief exit surveys with enrolled patients, in-depth interviews with a purposive subsample of enrolled patients, focus group discussions with the TB counsellors, and field observations [28, 29]. The in-depth interviews and focus group discussions followed semi-structured interview guide with open-ended and exploratory questions. We asked patients for their feedback on the counselling sessions (such as convenience, timing, quality of counselling, breadth of material, and helpfulness), and we asked counsellors for their feedback on the intervention as a whole (such as the overall implementation, pre-intervention training, health talks, counselling sessions, perception of patient and clinic staff response to the intervention, and suggestions for improvements). Qualitative data were collected by trained team members not directly engaged in counselling and audio-recorded. Data were transcribed, translated, de-identified, and thematically analyzed [30] (using Microsoft OneNote) inductively first, then deductively, to examine the barriers, facilitators, and contextual factors or circumstances that shaped intervention delivery (among TB counsellors) and intervention uptake (among patients). During this process, we learned how specific principles of motivational interviewing and social work practice were accepted by patients. Analyses were crosschecked (SL and AD) for inter-rater reliability, and enhanced analytic credibility and dependability [31, 32].

## Results

### Overview

**Fig. 1:**
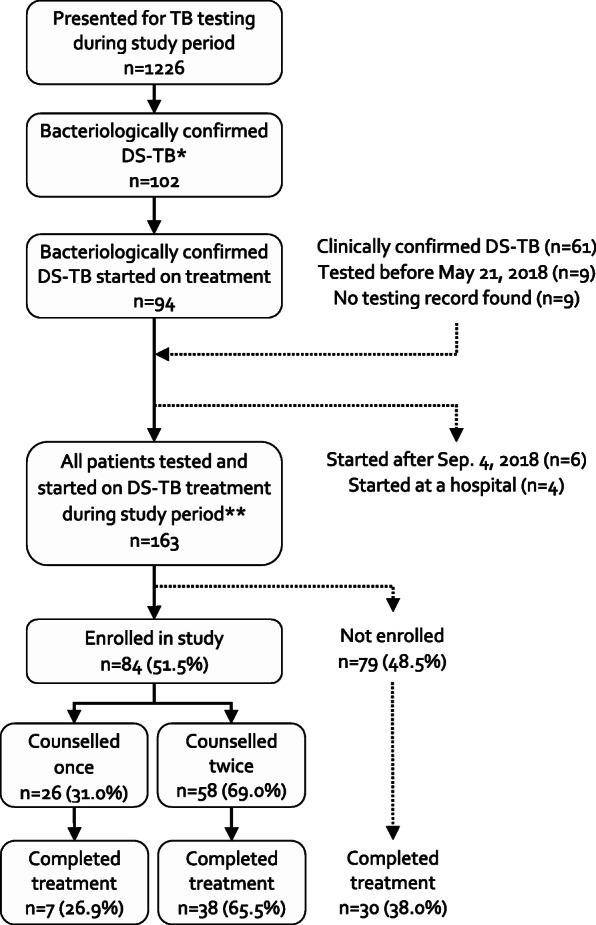
Patient flow chart of testing and treatment initiation at the study clinic during the study period (May 21 to Sep. 4, 2018, inclusive). *Excludes 6 RIF-resistant TB cases **Excludes 18 patients who were diagnosed elsewhere and transferred for treatment at the study site during the study period

Prior to patient enrollment, we included 14 clinic staff in the pre-study focus group discussion and trained 11 TB counsellors to deliver the intervention (Table 2). During the study period, there were: 1226 individuals who presented for TB testing at the clinic; 163 TB patients who started treatment; and 84 TB patients (42.3% female) who enrolled in our study (Fig. 1). There were no statistically significant differences (*p* > 0.10) in baseline patient characteristics (age, sex, HIV status, smear-positivity) between those enrolled and not enrolled in our study, except fewer HIV-positive patients were on ART at the start of TB treatment among those enrolled (*p* = 0.01) (Table 3). Of enrolled patients, 58 (69.0%) completed both counselling sessions; they were more likely to have a successful treatment outcome (*p* < 0.05) compared to those who only completed one session (Additional file 1:Table 2). Of those who completed both sessions, 57 (98.3%) completed exit surveys and 13 (15.5%) completed in-depth interviews.

**Table 2:** Study participants and activities

Activity/participant type	No. (%)
*Pre-study focus group with clinic staff*	*14 (100)*
Counsellors	7 (50)
Doctor or assistant doctor	2 (14.3)
Nurses	3 (21.4)
Administrative staff	2 (14.3)
*TB counsellor training*	*11 (100)*
Group 1 (May 21 – Jul 13)	7 (63.6)
Group 2 (Jul 14 – Sep 4)	4 (36.4)
*Enrolled patients*	*84 (100)*
Exit surveys	57 (67.9)
In-depth interviews	13 (15.5)

**Table 3:** Baseline patient characteristics and treatment outcomes stratified by study arms

Baseline patient characteristic	Study vs historical control period			Study period only		
	Historical control	Study	Chi-square p-value	Not enrolled in study	Enrolled in study	Chi-square p-value
*Baseline patient characteristics*						
Age, mean (SD)	37.0 (14.7)	35.7 (13.2)	0.45	37.3 (12.5)	34.1 (13.7)	0.12
Female (%)	38 (34.5)	69 (42.3)	0.24	37 (46.8)	32 (38.1)	0.33
HIV-positive (%)	78 (70.9)	114 (69.9)	0.97	56 (70.9)	58 (69.0)	0.93
On ART at start of treatment (%)	40 (51.3)	47 (41.2)	0.22	30 (53.6)	17 (29.3)	0.01
Previously treated* (%)	18 (16.4)	40 (24.5)	0.14	15 (19.0)	25 (29.8)	0.16
Smear-positive (%)	35 (31.8)	33 (20.2)	0.04	14 (17.7)	19 (22.6)	0.60
*Treatment outcomes*						
Success (Cured/completed)	50 (45.5)	75 (46.0)	0.16	45 (53.6)	30 (38.0)	0.27
Died	3 (2.7)	4 (2.5)		1 (1.2)	3 (3.8)	
Lost to follow-up	9 (8.2)	18 (11.0)		8 (9.5)	10 (12.7)	
Transferred out	26 (23.6)	50 (30.7)		24 (28.6)	26 (32.9)	
Not evaluated	22 (20.0)	16 (9.8)		6 (7.1)	10 (12.7)	

*Abbreviations*: *ART* Antiretroviral therapy, *SD* Standard deviation

Qualitative and quantitative findings are presented in tandem ahead, organized according to three phases of the study cascade: 1) study implementation; 2) testing and treatment initiation; and 3) study and treatment retention. Representative quotes are linked to participant type and, for patients, gender and age.

### Study implementation

#### Clinic engagement and limitations

TB counsellors were assigned to the clinic for at least 1 week prior to the study training. The opportunity allowed them to become familiar with clinic staff, establish rapport, and learn about the routine patient referral processes. Pre-study involvement of clinic staff helped to gain their support and buy-in during study implementation. TB counsellors reported that after the first week of getting accustomed to the intervention protocol, clinic staff were consistently cooperative, referred new patients for counselling, and took effort to share private rooms for counselling when needed.

“I think that approach helped them because they felt included; it did not appear to just come from the department of social work.” - TB counsellor.

On occasion however, counsellors were unable to find a private room, which resulted in delays or postponements. Furthermore, only about half of potentially eligible patients were enrolled in the study; they were missed because TB counsellors’ work hours were shorter than clinic hours (24/7), restricted to weekdays, and sometimes curtailed due to academic or other commitments.

#### Counsellors’ knowledge gaps

While none of the TB counsellors had prior training in TB, 5 (45.5%) had personal or family history of TB, and all of them gained significant knowledge after the study training. Their median TB knowledge score increased from 50 to 65% after training (Additional file 1: Table 3), and importantly, they became more aware of patient barriers to TB treatment and adherence (*p* = 0.01) (Additional file 1: Table 4), including: poverty, drug supply shortage in the clinic, traditional and religious beliefs, distance from the clinic and lack of information. All counsellors agreed the training reduced their anxieties around being near TB patients.

“The training at first was scary … when they mentioned that we will be dealing with patients with TB … we were afraid that what if we get infected. But as we get more information during training, we left feeling alright; we understood and being scared had decreased, but we were still scared.” – TB counsellor.

#### Proficiency and tedium of health talks

TB counsellors delivered health talks on 58 days at 5 different clinic wards (paediatric, maternity, primary care, dental and HIV), which took an average of 94.1 (± 42.4) minutes per day, including time for questions and answers, and several interruptions. They reported feeling increasingly comfortable delivering information about TB. However, after the first few weeks, counsellors felt the health talks were redundant. They experienced fatigue with daily talks and gradually reduced them to thrice weekly. Several counsellors independently sought opportunities to deliver health talks outside the clinic, such as during a health awareness campaign orchestrated by the Community Police Forum.

“Let’s say go outside in the community … Because at the clinic you end up talking to the same people.” – TB counsellor.

### TB testing and treatment initiation

#### Perceived patient engagement

**Fig. 2:**
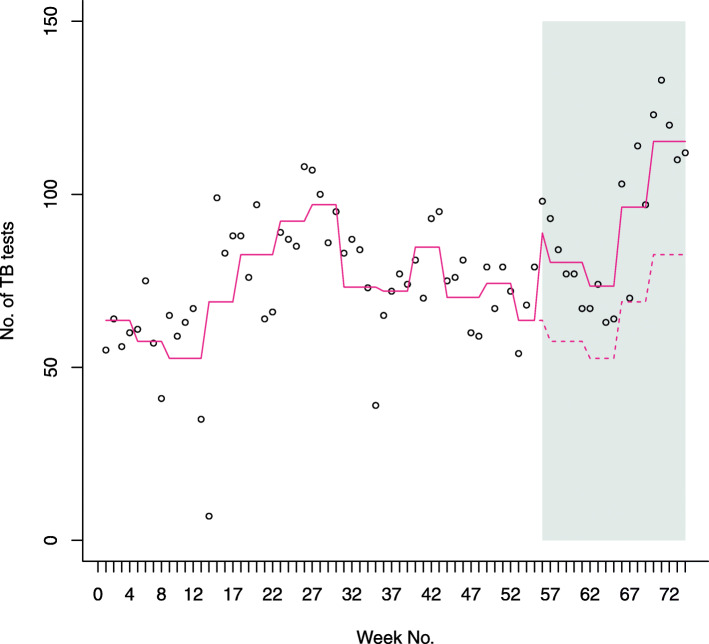
Plot showing the number of TB diagnostic tests performed weekly between May 2017 and September 2018 (represented by circles). Week 1 represents the first week of May 2017 and week 74 represents the last week of September 2018; the intervention started at week 56 and is represented by the grey shading. The solid red line represents the *monthly* trend in observed number of TB diagnostic tests, the dotted red line shows the expected number of TB diagnostic tests during the study period if the background, pre-study trend continued

The number of TB tests performed increased by 1.36-fold (95%CI 1.23 to 1.58) post-intervention, adjusting for the background seasonal trend (Fig. 2); the proportion tested positive remained similar (chi-square *p*-value = 0.62). Comparing the study period to historical control period, the probability of treatment initiation increased from 7.8 to 19.0%, representing an increase of 10.1% (95%CI 1.5 to 21.3%) after adjusting for potential confounders; median treatment delay decreased from 7.0 days to 4.5 days, a change of 2.5 days (95%CI 2.0 to 3.0 days) (Table 4 and Additional file 1: Table 5).

**Table 4:** Joint summary of main quantitative and qualitative findings

	Quantitative analyses	Qualitative themes
**Study implementation**	TB counsellors’ median pre-training and post-training TB knowledge score were 50% (IQR 7.5%) and 65% (IQR 17.5%). The median change in score was 12.5% (95%CI = 5.0 to 20.0%)^a^ Enrolled 51.5% (84 out of 163) of all TB patients who started treatment during the study period. Median duration of health talks (*n* = 58) was 94.1 (± 42.4) minutes	• Clinic engagement and limitations • Counsellors’ knowledge gaps • Proficiency and tedium of health talks
TB testing & treatment initiation*	Comparing the study period to the historical control period: - Number of diagnostic tests increased by 1.36 times (95%CI 1.23 to 1.58) (see Fig. 2 for observed and expected TB tests performed pre-and post-intervention)^b^ - Probability of treatment initiation increased from 7.8 to 19.0%; with an estimated increase of 10.1% (95%CI 1.5 to 21.3%) after adjusting for potential confounders^c^ - Median treatment delay decreased from 7.0 days to 4.5 days, a change of 2.5 days (95%CI 2.0 to 3.0 days)^d^	• Perceived patient engagement • Difficult patient queries
Treatment retention*	Probability of treatment completion was similar during the study period (45%) and the historical control period (46%). There was an estimated increase during the study period of 4.4% (95%CI −7.3 to 16.0%) after adjusting for baseline confounders^e^ Among those enrolled, 26 (31%) received only the first of two study counselling sessions.	• Improved treatment self-efficacy • Alleviation of anxiety, fears and perceived stigma • Barriers to treatment and counselling

^a^Estimated using a Wilcoxon rank-sum test

^b^Rate ratio comparing number of TB tests performed during the study period to the historical control period estimated via a quasi-Poisson regression model, including a dummy indicator variable for the intervention and a fixed effect for calendar month to account for background seasonal trend.

^c^Estimate adjusted for age, sex and calendar month using a multivariate binomial regression model with an identity link.

^d^Confidence interval of the difference in medians estimated using the adjusted bootstrap percentile (BCa) method.

^e^Based on an intention-to-treat analysis comparing all patients who started treatment during the study period versus the historical control period, using a binomial regression with an identity link, and adjusted for the following baseline characteristics: age, sex, smear status, and HIV and ART status

These findings were supported by the TB counsellors’ perception of patient interest and engagement during health talks, demonstrated by their frequent questions and exchange of personal experiences.

“I remember a day we were doing a presentation where old people sit … there was a brother who was arguing and saying TB is not curable, and [a woman] stood up and said; ‘I, my child, I had MDR [and] look, I am alive.’ So I was very happy that day because there is evidence, a patient saying for herself that ‘I was in hospital for six months and I was cured and TB is curable.’ You could see the brother calming down, starting to believe because of hearing from a person who was in treatment.” – TB counsellor.

TB counsellors believed the health talks encouraged people to get tested for TB and reinforced the importance of timely treatment. They also remarked on how they, as well as patients who heard the talks, were encouraged to share what they learned about TB with others.

“You see that the person wants to get more information and you see that the information that he is getting he does not want to keep it for himself; he wants to pass it on to another person so they want to understand well.” – TB counsellor.

#### Difficult patient queries

TB counsellors grew more confident fielding patients’ questions over time and appreciated being mentored by the study team. On several occasions during the first couple weeks of the study, counsellors had to seek assistance from study team members with more extensive knowledge about TB to field specific TB testing or treatment-related questions, especially ones about drug-resistant TB – a topic that was not comprehensively covered during the one-week training. This led some counsellors to worry that their competence or trustworthiness was being challenged.

“Sometimes a problem is that there are patients it [who] are educated, sometimes they have studied the course you are doing [on TB] more than you – you only did it for three days – and he knows more. I came across with that challenge, but I was with one of our colleagues, so I ended up asking her to please answer for me, because the way he was directing the questions, he was challenging me.” - TB counsellor.

Over time, TB counsellors became more confident in their knowledge and were able to handle challenging questions and situations independently. An example given by one counsellor was how she knew what to say and do when faced with an emotional patient who had just been diagnosed with both HIV and TB.

“[The patient] was crying having found out that she is positive and TB was found. So it means that she got it all in one time. We had to offer counselling that on one side it is HIV, it is treatable if you take your pills the right way, before we even speak about TB...[but] the training helped because I applied the skills that I was taught, that if a patient it is like this, how you should approach it.” – TB counsellor.

### Treatment retention

#### Improved treatment self-efficacy

There was a 4.4% (95%CI − 7.3 to 16.0%) increase, adjusting for baseline confounders, in the probability of TB treatment completion during the study period (46%) compared to the historical control period (45%); patients who were enrolled in our study had an 11.2% (95%CI − 1.6 to 23.8%) higher probability of completing treatment (42%), compared to all other patients who started treatment in both the study and historical control period (54%) (Table 4 and Additional file 1: Table 8).

Several study patients said the counsellors helped them understand the importance of treatment, and further instilled confidence that they could finish the course. Counsellors in turn believed this reduced the workload of clinic staff by preventing losses to follow-up and repeat treatment.

“The counsellors were friendly, they showed care in me, and believed in me that I will complete treatment.” – male TB patient, 43.

“Because I saw improvement in how people adhere to medication through counseling, that helps the clinic staff because they … do not have to go back and treat the person for TB [again], so there was improvement in the hospital as a whole.” – TB counsellor.

TB counsellors attributed the perceived improvements in adherence to increased patient knowledge and understanding about TB treatment, trust and confidence built between patients and counsellors, and attention to patient-centred solutions to address adherence challenges.

#### Alleviation of anxiety, fears and perceived stigma

All patients who completed the exit survey found the sessions to be helpful (Additional file 1: Table 6). They especially appreciated being given an opportunity to ask questions and be listened to without judgment.

“The counsellor was very helpful and gave information, gave a chance to ask question … it was not pleasant at all to hear that I had TB, but the counselling was helpful, it helped the me to accept the situation and gave me the hope to live again.” – female TB patient, 48.

Several patients expressed feelings of denial or hopelessness during counselling. They described how the sessions helped them cope with the initial shock of being diagnosed and assuaged some of their concerns and stigmas about TB.

“It helped me to realize that TB can affect everyone; before I had a perception that people who get TB stay in informal settlements, attending sessions changed my way of thinking.” – male TB patient, 56.

Counsellors confirmed that many patients were shocked by their diagnosis, particularly if they had little prior knowledge about TB. They emphasized the importance of giving space for such patients to process the news, and how this allowed patients to contemplate and ask more focused questions about the treatment process.

“A social worker is able to give you time to listen to you, [and discuss] how you will overcome these problems that you say you have; we meet each other halfway, we discuss it … But a nurse won’t have time, the queue is long … she cannot sit with a patient for 45 minutes.” – TB counsellor.

TB counsellors thus filled an important gap, which had been identified by clinic staff during the pre-study focus group discussion, namely, the lack of time and opportunity to properly educate and prepare TB patients at the start of treatment.

#### Barriers to treatment and counselling

During the pre-study focus group discussion, clinic staff brought up the following main challenges to retention-in-care: inadequate patient preparation and education; denial of diagnosis and treatment; stigma; initial shock and weakness (as hindering education); transient catchment population; difficulties tracing homeless patients; and job suspension (Table 5). The counselling sessions addressed many of these barriers, as discussed earlier. However, the intervention was unable to address specific needs of homeless, transient or unemployed patients. Indeed, nearly one-third of enrolled patients were transferred to other clinics and lost from study follow-up (Table 3).

**Table 5:** Themes from focus group discussion with clinic staff TB adherence and retention

Theme	Representative quotes from health care workers
Inadequate patient preparation and education	“Now I am rushed … I am teaching you bits and pieces and say you will see the rest on the paper at home because I am rushing for the queue outside.”
	“It seems better at HIV because, you know, HIV like they have a lot of time than us, they have time for testing, they are taught in classes, and they are prepared before they start pills; with TB, you find out today that you have TB, you start taking pills today.”
Stigma	“Maybe they will be afraid that they have TB because it will be said if you have TB you will infect [others] while working, in that way it makes them stigmatized.”
Initial shock and weakness (as hindering education)	“Others you even finish talking to them and they are just shocked, they can’t even hear what you are saying.”
	“Health education is given to TB patients but because they come weak or have errands, some are disorientated”
Denial of diagnosis and treatment	“They just want cough mixture and antibiotics … so now for them to come back for you to give them medication - that’s going to be chronic - is a problem, that’s the biggest problem … They are not here thinking they will get TB, they just are here for a quick fix.”
Transient catchment population	“We are in town … where they get their buses, taxis and all those things, so a person walks in and they are not from our catchment area so maybe they are from Ianskop or wherever … so we have done screening and we see that they have signs and symptoms … they are not going to come back because they are not coming here for that.”
Difficulties tracing homeless patients	“There are many people who are homeless, some are living in the streets, some in informal settlements so you find that the patient will come in when they are very sick, they come and take medication … As soon as they feel they are a bit better they will go back to their lives of hustling, so they go back and hustle and it becomes difficult for us to even trace them.”
Job suspension	“We have a problem with employers … employers, once they find out they have TB they stop them from work, so they need letters from doctors that will say whether the person can continue with working or not.”

In recognizing the initial shock or weakness experienced by patients, counsellors tried to be flexible and accommodated patients’ needs by moving sessions to a future appointment, when patients felt more ready to learn about their treatment. They also tried to see patients while they were waiting in queue to be seen by the TB staff, thereby reducing the time spent at the clinic.

“I was still in pain and thinking about my family’s situation and hunger.” – male TB patient, 47.

“Sometimes [the patient] came to the clinic for his date, but he was tired because he arrived in the morning and was in queues; he has gotten his pills why not go home.” – TB counsellor.

Most (90%) patients who completed an exit survey found the sessions to be conveniently timed. However, 16% found them to be too long (Additional file 1: Table 7). The average duration of each counselling session was similar; approximately 20 min (Additional file 1: Table 6).

Patients who were experiencing no treatment challenges felt the second session to be redundant. However, counsellors felt this session was crucial to reinforcing the importance of adherence at a time when patients might feel recovered and de-prioritize adherence.

“Once [the patient] has been diagnosed with TB, he already has the drive to that ‘I have to cure this’, he is listening to everything you are saying. But when it’s coming to three months, when they are starting to go back to normal … it is where they start to be lost to follow-up, it’s where they go back and become sick.” – TB counsellor.

Acceptability of the counselling sessions for patients was also influenced by some of the implementation or feasibility issues experienced in the study. For example, as mentioned previously, lack of private spaces at the clinic and scheduling issues with the counsellors sometimes led to delays, postponements or even cancellations of counselling sessions. This could compromise the acceptability of the intervention as it could render the counselling sessions less convenient or efficient for patients.

## Discussion

Our study provided evidence that a TB literacy and treatment counselling intervention was feasible, acceptable, and had a significant impact on TB testing and treatment initiation rates, as well as a modest impact on retention-in-care in a resource-limited setting with a high burden of TB and HIV. We used a unique participatory approach by involving site health care workers in the development of the intervention, which facilitated their buy-in and support during the study. Furthermore, we capitalized on the availability of social work students to provide TB literacy education and treatment counselling, which helped fill an important service gap at the study clinic. This model could likely be generalized to other settings through engagement with schools of social work.

The group health talks at the clinic seemed to have increased the general clinic population’s interest in TB testing, and thus helped increase TB testing uptake during the study period. However, the counsellors described the health talks as being quite repetitive and tedious. To circumvent the tedium, the counsellors reduced the frequency of the talks and sought speaking opportunities outside the clinic. These endeavours helped diversify the counsellors’ experience as well as their audience. The TB counselling sessions addressed some key treatment literacy and adherence challenges identified by health care workers at the study clinic. To this end, the intervention gave patients an opportunity to ask questions, be heard, and resolve misinformation, through dedicated time with a unique cadre of provider that had a social work skillset and substantive knowledge about TB. Allowing sufficient time for patient-counsellor interactions builds trust and rapport, which could influence patient adherence and outcomes [33]. Outsourcing counselling away from the primary TB nurse allowed for some flexibility in the routinization of counselling sessions to accommodate the schedules of patients, many of whom were still coming to terms with their diagnosis.

Prior to the intervention, due to time and resource constraints, no treatment education nor counselling was being provided routinely to TB patients. The improvements to patient outcomes could be attributed to the education and counselling, but also potentially to the modest increase in patient time and encounters; a couple systematic reviews have noted that increasing frequency and duration of patient encounters alone could improve outcomes [8, 34]. Nonetheless, our intervention was not positioned to address all challenges to retention-in-care, such as dealing with a transient catchment population.

Several important limitations may have affected our findings. First, study enrollment and retention were impeded by counsellor absenteeism (often due to other academic commitments) and space constraints at the site. Some of this is owing to the pilot nature of the study as well as recruitment of ‘volunteer’ student interns who had competing academic priorities. Together with counsellor fatigue, this may have led to waning interest to contribute to the study. Reducing the frequency of health talks relieved some of that fatigue. Second, although the training greatly improved TB knowledge among our counsellors, important gaps were revealed during their delivery of health talks pointing to the need for refresher trainings. Third, our data collection period was short and included a small study sample, which affected the statistical power of our study findings and potentially the generalizability of our study. Fourth, due to time and resource constraints, we were unable to conduct a post-intervention focus group discussion with clinic staff to assess their perspectives on the intervention. Nonetheless, the observations and feedback from the study counsellors suggested an overall positive reception of the intervention by clinic staff. Finally, due to the group healhe highly transient catchment population, up to one-third of patients who started treatment during the study period were transferred out and lost to study follow-up, which likely diluted the impact of the counselling sessions on treatment outcomes during the study period.

The limitations to our pilot study provide valuable lessons for scaling up the intervention for broader delivery in South African primary care clinics. Several major implementation and feasibility issues plagued this pilot, which would likely be common problems if the intervention were offered at other similar settings. These include the lack of space and private rooms for counselling sessions, tedium and burn-out among counsellors, and scheduling or timing conflicts between room, counsellor and patient availabilities. We have proposed some possible solutions to deal with these issues, such as reducing frequency of health talks and adding external outreach opportunities to reduce tedium. But importantly, for an intervention such as this to work, it should ideally be fully integrated within the clinic with a constant rotation of trained interns such that all patients are offered at least first-time counselling. This was not achieved in our study and unfortunately meant about half of the new TB patients at the clinic were missed.

The evidence from this study nonetheless makes important contributions to developing patient-centred interventions to improving retention along the cascade of TB care. To our knowledge, this is the first study that utilizes student social worker skills to develop patient literacy about TB testing and treatment. We showed it is possible to train junior social work students to provide essential TB treatment education and counselling, thereby increasing TB testing and treatment uptake, and modestly improving patient retention-in-care. This would be particularly relevant for other clinics that routinely host social work interns (or interns in related social or health care fields) and currently struggle to provide routine treatment counselling or education to their patients. Also explicitly used motivational interviewing techniques, which appeared to boost patients’ confidence in their ability to adhere (i.e., self-efficacy), and substantiates evidence on treatment adherence interventions in HIV [26]. Our study confirms the gains in TB treatment outcomes that may be achieved with dedicated TB counselling and psychoeducational support [35–36]. It builds on the experience in Cape Town, South Africa, where the provision of early treatment counselling allows patients to receive community-based, instead of clinic-based, DOT [21]. Lastly, this study contributes to sparse evidence on reducing initial or pre-treatment losses to follow-up, that is, to increase the treatment initiation rate among individuals who test positive for TB.

## Conclusions

Our pilot treatment literacy and counselling intervention was feasible, acceptable, and increased TB testing, treatment initiation, and completion rates at a busy primary care clinic. Our study showed that routine health talks delivered at a busy clinic could improve TB testing and treatment initiation rates and may be feasibly performed by trained non-medical counsellors, thereby exemplifying a model for task shifting. This is a significant contribution given the large estimates of initial losses of follow-up in countries such as South Africa, as well as the demand for strategies that may be implemented within the existing resources of health systems.

## Supplementary Information

**Additional file 1..**

## Data Availability

The datasets used and/or analyzed during the current study are available from the corresponding author on reasonable request.

## Abbreviations

ART: Antiretroviral therapy
CI: Confidence interval
DOT: Directly observed therapy
FGD: Focus group discussion
KAP: Knowledge, attitudes and practices
KZN: Kwazulu-Natal
TB: Tuberculosis
