# Supplementary material for: Acceptability, feasibility, and impact of a pilot tuberculosis literacy and treatment counselling intervention: a mixed methods study
Source: BMC Infect Dis. 2021 May 18;21:449. doi: 10.1186/s12879-021-06136-1 (PMC8132373; doi:10.1186/s12879-021-06136-1)
Supplement: Supplementary file 1 — Additional file 1.. [file 12879_2021_6136_MOESM1_ESM.docx]

APPENDIX

Contents

[Box 1. Summary of intervention framework 2](#_Toc47045203)

[Box 2. Patient adherence plan 3](#_Toc47045204)

[Table 1. TB counsellor training schedule 4](#_Toc47045205)

[Box 3. TB counsellor knowledge, attitudes and practice (KAP) survey 5](#_Toc47045206)

[Box 4. Brief patient exit survey 9](#_Toc47045207)

[Table 2. Baseline patient characteristics and treatment outcomes stratified by number of counselling sessions received among enrolled study patients 10](#_Toc47045208)

[Table 3. Responses to TB KAP survey knowledge questions pre- and post-training* (n=11) 11](#_Toc47045209)

[Table 4. Responses to TB KAP survey attitude questions pre- and post-training* (n=11) 13](#_Toc47045210)

[Table 5. Treatment initiation after bacteriologically confirmed TB diagnosis during study and historical control periods* 15](#_Toc47045211)

[Table 6. Details on counselling sessions 15](#_Toc47045212)

[Table 7. Patient responses to Yes/No questions on the exit survey (n=57) 16](#_Toc47045213)

[Table 8. Comparing the proportion of patients who successfully completed TB treatment 16](#_Toc47045214)

| Box 1. Summary of intervention framework Outline  The intervention has 3 stages:   1. Pre-diagnosis: health talks delivered to all patients waiting for services at the clinic 2. Post-diagnosis: 1^st^ counselling session with patients starting DS-TB treatment 3. During treatment: 2^nd^ counselling session 1 to 2 months after treatment initiation   Study counsellor tasks and responsibilities  In general, the health talk will be expected to last 10 minutes each, and the individual counselling sessions will last 20 minutes, and no longer than 45 minutes. Study counsellors will attempt to coincide the 2^nd^ one-to-one counselling session with the first or second monthly clinic visits. If a participant is unable to complete the session on the clinic visit, study staff will schedule a visit to complete the study procedure.  The goals of the counselling sessions are to enhance and support motivation for: returning to clinic for test results; initiating timely TB treatment; and adherence to and completion of TB treatment. During individual counselling sessions, study counsellors should identify and address as far as possible obstacles or challenges relating to adherence, and or treatment; to connect participants as is appropriate with referrals and other services; and to communicate to participant goals and upcoming elements of the intervention.  Study coordinator and counsellors will obtain the date for the next clinic visit from the participant or from the clinic staff. Using this information, the staff will determine the number of expected study visits for each clinic day. The counsellors will meet monthly or as required with the study social work supervisor to review cases and receive input and support.  General guidelines for counselling sessions  The sessions will employ a patient-centred, motivational interviewing (MI), problem solving approach. Patient-centred means that the counselling session will focus specifically on the medical, social and support needs of each patient. The motivational interviewing approach is defined as “empathic, person-centered counseling approach that prepares people for change by helping them resolve ambivalence, enhance intrinsic motivation, and build confidence to change.” The patient will identify barriers to medication adherence and retention in care, and patient and counselor will use this to develop goals for treatment and on-going dialogue to support the patient’s progress. The counselor will use open-ended questions, affirmation, reflective listening, and summary reflections to guide counseling sessions, with special attention to change statements. Not all patients will encounter barriers or desire counseling. These patients should view the counselor as a supporter at the clinic who is concerned about their well-being and continued progress.  Referrals to additional services  If a patient needs to be referred for additional social services, the study counsellor will complete a referral following clinic protocol. The patient can also be brought to the social work supervisor to discuss referrals.  Recordkeeping  A record of each health talk will be kept that includes: time and date of talk, approximate number of individuals the talk was delivered to, and any events that occurred during or as a result of the health talk (e.g. a patient came to ask questions). A record of each counselling session will also be kept that includes: PID, date, brief counselling notes, any referrals made (type and description), checklist of patient issues. Each patient will have their own folder containing all their records.  Health Talk and Counselling Guides  Health Talks and counselling sessions are unscripted; however, guides are provided for each counselling initial sessions. While unscripted, each session should generally follow these guides. |
| --- |

| Box 2. Patient adherence plan **PATIENT ADHERENCE PLAN Date ______________**  My motivation to complete treatment:  My plan to deal with side effects is:  My supporter is who, and why:  How will I remember to take my weekend medication:  Where will I keep my medication:  My reminder tools are:  How do I protect my family from TB infection?      Dealing with traditional medicines/alcohol/drugs:  Managing unplanned trips:  Additional notes: |
| --- |

| Table 1. TB counsellor training schedule | |
| --- | --- |
| Day 1 | - Introduction of study - Obtain informed consent - Complete pre-training KAP survey - Outline study logistics |
| Day 2 | - Review basic HIV/TB counselling skills - Introduce patient-centred care - Discuss HIV/TB-related stigma |
| Day 3 | - Discuss common TB treatment adherence challenges - Brainstorm and discuss adherence plans and strategies to address barriers - Role-play counselling scenarios with different adherence challenges - Intoroduction on TB transmission and pathogenesis, treatment, and drug resistance - Develop and practice TB health talks |
| Day 4 | - Distribute and review intervention toolkit (counselling logs and guides, patient consent and enrolment forms) - Review external health and social services for patient referrals - Detailed education on TB drugs and side effects, adverse events management and monitoring, and drug resistance |
| Day 5 | - Role-play and practice counselling sessions - Review and discuss ethics and boundaries, roles and responsibilities - Complete post-training KAP survey |

| Box 3. TB counsellor knowledge, attitudes and practice (KAP) surveyPersonal questions  1. Have you ever received tuberculosis-specific training before the current study?    1. Yes, in the past 12 months (before this study)    2. Yes, more than 12 months ago (before this study)    3. No 2. Have you ever been sick with tuberculosis?    1. Yes, before this study    2. Yes, during this study    3. No 3. Has someone you are in close contact with ever been sick with tuberculosis (for example: family members, spouse, friends/peers, etc.)?    1. Yes, before this study    2. Yes, during this study    3. No  TB knowledgeTB transmission  1. What is the causative agent of tuberculosis?    1. Mycobacterium tuberculosis    2. Mycobacterium avium    3. Mycobacterium pneumoniae    4. Mycobacterium leprae 2. Is tuberculosis a transmissible disease?    1. Yes    2. No 3. How does tuberculosis spread?    1. Sexually    2. By droplet spread in the air    3. By direct contact (skin-to-skin, kissing, etc,)    4. By sharing needles    5. It is not transmissible 4. Which group of people below is at highest risk of developing tuberculosis?    1. Patients with COPD    2. Patients with HIV    3. Males    4. People living in tropical areas 5. Briefly describe preventive measures you can take when you are dealing with a tuberculosis patient or someone who might have active tuberculosis  TB diagnosis  1. Please list the common symptoms of pulmonary tuberculosis: 2. What is the standard diagnostic tool for tuberculosis at this clinic? 3. Blood culture 4. Skin test 5. GeneXpert test 6. Culture 7. Sputum smear microscopy 8. TB diagnosis in children is more difficult than in adults? 9. yes 10. no   Briefly explain your answer:   1. How long is the expected waiting time for the TB test results? 2. 24 hours 3. 48 hours 4. 3 to 5 days 5. 1 week  TB treatment  1. Is tuberculosis a curable disease? 2. Yes 3. No 4. How long is standard treatment for drug-sensitive tuberculosis? 5. 2 months 6. 6 months 7. 9 months 8. 12 months 9. How long is the intensive phase of treatment for drug-sensitive tuberculosis?    1. 1 month    2. 2 months    3. 3 months    4. 4 months 10. How many drugs are used in the intensive phase of treatment for drug-sensitive tuberculosis? 11. 1 12. 2 13. 3 14. 4 15. How long is the continuation phase of treatment for drug-sensitive tuberculosis?     1. 1 month     2. 2 months     3. 3 months     4. 4 months 16. How many drugs are used in the continuation phase of treatment for drug-sensitive tuberculosis?     1. 1     2. 2     3. 3     4. 4 17. Briefly describe what directly observed treatment (DOT) is: 18. What is multi-drug resistant tuberculosis? 19. Tuberculosis that is resistant to Isoniazid and Rifampicin 20. Tuberculosis that is resistant to any one tuberculosis medication 21. Tuberculosis that is resistant to all tuberculosis medications 22. Tuberculosis that is resistant to Pyrazinamide and Ethambutol 23. In which group of people is multi-drug resistant tuberculosis most likely to occur? 24. In people with HIV 25. In people who have been exposed to other tuberculosis patients 26. In people who have been treated for tuberculosis before 27. In people who have never had tuberculosis before 28. What is the minimum duration of treatment for multi-drug resistant TB?     1. 6 months     2. 9 months     3. 12 months     4. 18 months 29. When should the first follow up sputum sample be carried out following the commencement of treatment of a confirmed case of tuberculosis? 30. 1 month after the commencement of treatment 31. 2 months after the commencement of treatment 32. 3 months after the commencement of treatment 33. 6 months after the commencement of treatment 34. What is the major element to assess tuberculosis treatment cure? 35. Skin test 36. Chest X-ray 37. Sputum 38. All of the above 39. If a patient’s TB culture test results are negative, are they still infectious?     1. Yes     2. No     3. Cannot answer without more information 40. What are the consequences of incomplete treatment? 41. Development of resistant tuberculosis 42. Failure to fully cure the disease 43. Further transmission of the disease 44. All of the above 45. List several common side effects of standard treatment for drug-sensitive tuberculosis:  TB-related attitudes Please circle the response that most applies to you. [The choices for each statement were: Strongly agree; Agree; Neutral; Disagree; Strongly Disagree]   1. Finding every new case of tuberculosis is essential for control of the disease 2. Community engagement is essential for the control of the disease 3. There is a substantial increase in treatment completion rates if direct observed treatment is used 4. There is a stigma associated with tuberculosis in Kwazulu-Natal. Briefly explain your answer. 5. The way you interact with tuberculosis patients can contribute to any existing stigma. Briefly explain your answer. 6. Tuberculosis as a disease has more stigma associated with it than HIV 7. Directly observed treatment (DOT) is more effective than education in ensuring patient adherence to TB treatment 8. In Kwazulu-Natal, the general population is aware of the tuberculosis services that are available 9. Public awareness regarding tuberculosis as a health problem in Kwazulu-Natal is adequate. Briefly explain your answer. 10. Multi-drug resistant tuberculosis is a problem in Kwazulu-Natal 11. Traditional or alternative medicine assists in wellbeing of tuberculosis patients 12. Standard first-line treatment for drug-sensitive tuberculosis is accepted by patients 13. In Kwazulu-Natal, there are many barriers to tuberculosis treatment. Briefly explain your answer. 14. Making people with possible/confirmed pulmonary tuberculosis wear masks in the clinic is acceptable 15. Teaching tuberculosis patients cough hygiene is not important 16. Infection control is an important means to prevent contracting tuberculosis 17. I have been seriously concerned I have had tuberculosis 18. I should know whether I’ve got or had tuberculosis 19. If I contracted tuberculosis, I would be allowed to continue working in my current capacity 20. My employer would maintain confidentiality if I were to contract tuberculosis 21. I should know my own HIV status. Briefly explain your answer.  TB practices  1. List the clinic members involved in the healthcare team in TB patient-centred care 2. Briefly describe the purpose of the clinic treatment card 3. Describe three scenarios in which you would refer a patient to another clinic staff or to another healthcare worker outside the clinic 4. If during a counselling session, a TB patient tells you they have not been taking their pills, how would you respond and what would you do? |
| --- |

| Box 4. Brief patient exit survey  1. Did you find the counselling sessions helpful? 2. Yes 3. No   Briefly explain:   1. Did you like your counsellor(s)? 2. Yes 3. No   Briefly explain:   1. Did the counselling sessions take too much time?    1. Yes    2. No 2. Were the counselling sessions at a suitable/convenient time for you?    1. Yes    2. No   If no, briefly explain:   1. Did you feel comfortable asking questions in your counseling sessions?    1. Yes    2. No 2. Did you feel comfortable talking about your problems with the counsellor?    1. Yes    2. No 3. Did the counselling sessions make you feel more confident talking about your TB with loved ones (friends, family, peers)?    1. Yes    2. No 4. Were you happy with the counsellor’s answers to your questions?    1. Yes    2. No 5. Are you confident you will finish 6 months of treatment?    1. Yes    2. No 6. Did the counselling sessions make you feel more confident about finishing treatment?    1. Yes    2. No 7. Which counselling session did you like the most?    1. First (after getting tested)    2. Second (when I started treatment)    3. Third (when I had taken treatment for more than one month)   Briefly explain why:   1. Which counselling session did you like the least?    1. First (after getting tested)    2. Second (when I started treatment)    3. Third (when I had taken treatment for more than one month)   Briefly explain why:   1. What did you like about the counselling? 2. What did you not like about the counselling? |
| --- |

| Table 2. Baseline patient characteristics and treatment outcomes stratified by number of counselling sessions received among enrolled study patients | | | |
| --- | --- | --- | --- |
|  | **Counselled once (n=26)** | **Counselled twice (n=58)** | **Chi-square  p-value** |
| *Baseline patient characteristics* | | | |
| Age, mean (SD) | 34.6 (14.9) | 38.5 (11.2) | 0.24 |
| Female (%) | 10 (38.5) | 22 (37.9) | 1 |
| HIV-positive (%) | 18 (69.2) | 40 (69.0) | 1 |
| On ART at start of treatment (%) | 6 (33.3) | 11 (27.5) | 0.89 |
| Previously treated* (%) | 4 (15.4) | 21 (36.2) | 0.09 |
| Smear-positive (%) | 4 (15.4) | 15 (25.9) | 0.44 |
| Unemployed (%)** | 10 (38.5) | 23 (39.7) | 1 |
| Lives in an informal settlement (%) | 15 (57.7) | 35 (60.3) | 1 |
| Secondary education  Completed (%)  Incomplete (%)  None (%) | 14 (53.8) 8 (30.8) 4 (15.4) | 32 (55.2) 12 (20.7) 14 (24.1) | 0.49 |
| Smoking status  Current (%)  Former (%)  Never (%) | 6 (23.1) 5 (19.2) 15 (57.7) | 17 (29.3) 5 (8.6) 36 (62.1) | 0.37 |
| Alcohol consumption  Daily (%)  Less than daily (%)  None (%) | 1 (3.8) 4 (15.4) 21 (80.8) | 2 (3.4) 10 (17.2)  46 (79.3) | 1 |
| *Treatment outcomes* | | | |
| Success (Cured/completed) | 7 (26.9) | 38 (65.5) | 0.01 |
| Died | 1 (3.8) | 0 |  |
| Lost to follow-up | 4 (15.4) | 4 (6.9) |  |
| Transferred out | 12 (46.2) | 12 (20.7) |  |
| Not evaluated | 2 (7.7) | 4 (6.9) |  |

*Defined as a patient registered as retreatment after cure or loss to follow-up in the TB patient register
**Other employment status included: employed (full or part-time), self-employed, student and pensioner

| Table 3. Responses to TB KAP survey knowledge questions pre- and post-training* (n=11) | | | |
| --- | --- | --- | --- |
| **Question** | **Pre-training**  **(No. who selected response)** | **Post-training (No. who selected response)** | **Fisher’s exact test p-value** |
| General Knowledge | | |  |
| What is the causative agent of TB? | | | |
| **Mycobacterium tuberculosis** | 10 | 11 | 1.0 |
| Mycobacterium avium | 0 | 0 |  |
| Mycobacterium pneumoniae | 0 | 0 |  |
| Mycobacterium leprae | 1 | 0 |  |
| Is TB a transmissible disease? | | | |
| **Yes** | 10 | 10 | 1.0 |
| No | 1 | 1 |  |
| How does TB spread? | | | |
| Sexually | 0 | 0 | 1.0 |
| **By droplet spread in the air** | 11 | 11 |  |
| By direct contact (skin-to-skin, kissing, etc,) | 0 | 0 |  |
| By sharing needles | 0 | 0 |  |
| It is not transmissible | 0 | 0 |  |
| Which group of people below is at highest risk of developing TB? | | | |
| Patients with COPD | 1 | 0 | 1.0 |
| **Patients with HIV** | 9 | 10 |  |
| Males | 0 | 0 |  |
| People living in tropical areas | 1 | 1 |  |
| ***Diagnostics and testing*** | | |  |
| What is the standard diagnostic tool for tuberculosis at this clinic? | | | |
| Blood culture | 1 | 0 | 0.005 |
| Skin test | 1 | 0 |  |
| **GeneXpert test** | 0 | 7 |  |
| Culture | 0 | 0 |  |
| Sputum smear microscopy | 8 | 4 |  |
| TB diagnosis in children is more difficult than in adults? | | | |
| **Yes** | 10 | 11 | 1.0 |
| No | 1 | 0 |  |
| How long is the expected waiting time for the TB test results? | | | |
| 24 hours | 0 | 0 | 0.64 |
| **48 hours** | 2 | 0 |  |
| 3 to 5 days | 7 | 6 |  |
| 1 week | 2 | 3 |  |
| ***Treatment & management*** | | |  |
| Is tuberculosis a curable disease? | | | |
| **Yes** | 11 | 10 | 1.0 |
| No | 0 | 0 |  |
| How long is standard treatment for drug-sensitive tuberculosis? | | | |
| 2 months | 0 | 1 | 1.0 |
| **6 months** | 10 | 10 |  |
| 9 months | 0 | 0 |  |
| 12 months | 1 | 0 |  |
| How long is the intensive phase of treatment for drug-sensitive tuberculosis? | | | |
| 1 month | 4 | 0 | 0.008 |
| **2 months** | 3 | 9 |  |
| 3 months | 3 | 0 |  |
| 4 months | 1 | 2 |  |
| How many drugs are used in the intensive phase of treatment for drug-sensitive tuberculosis? | | | |
| 1 | 3 | 3 | 0.12 |
| 2 | 2 | 2 |  |
| 3 | 4 | 0 |  |
| **4** | 2 | 6 |  |
| How long is the continuation phase of treatment for drug-sensitive tuberculosis? | | | |
| 1 month | 0 | 0 | 0.49 |
| 2 months | 5 | 2 |  |
| 3 months | 1 | 2 |  |
| **4 months** | 5 | 6 |  |
| How many drugs are used in the continuation phase of treatment for drug-sensitive tuberculosis? | | | |
| 1 | 0 | 1 | 0.63 |
| **2** | 6 | 4 |  |
| 3 | 3 | 2 |  |
| 4 | 2 | 4 |  |
| What is multi-drug resistant tuberculosis? | | | |
| **Tuberculosis that is resistant to Isoniazid and Rifampicin** | 0 | 4 | 0.16 |
| Tuberculosis that is resistant to any one tuberculosis medication | 1 | 1 |  |
| Tuberculosis that is resistant to all tuberculosis medications | 6 | 5 |  |
| Tuberculosis that is resistant to Pyrazinamide and Ethambutol | 3 | 1 |  |
| In which group of people is multi-drug resistant tuberculosis most likely to occur? | | | |
| In people with HIV | 5 | 2 | 0.44 |
| In people who have been exposed to other tuberculosis patients | 2 | 4 |  |
| **In people who have been treated for tuberculosis before** | 4 | 5 |  |
| In people who have never had tuberculosis before | 0 | 0 |  |
| How long is standard treatment for MDR-TB? | | | |
| 12-15 months | 6 | 3 | 0.22 |
| **18-24 months** | 1 | 0 |  |
| 28-32 months | 1 | 5 |  |
| 36 months | 3 | 3 |  |
| When should the first follow up sputum sample be carried out following the commencement of treatment of a confirmed case of tuberculosis? | | | |
| 1 month after the commencement of treatment | 8 | 6 | 0.75 |
| **2 months after the commencement of treatment** | 0 | 2 |  |
| 3 months after the commencement of treatment | 2 | 2 |  |
| 6 months after the commencement of treatment | 1 | 1 |  |
| What is the major element to assess tuberculosis treatment cure? | | | |
| Skin test | 0 | 0 | 0.82 |
| Chest X-ray | 1 | 1 |  |
| **Sputum** | 3 | 5 |  |
| All of the above | 7 | 5 |  |
| If a patient’s TB culture test results are negative, are they still infectious? | | | |
| Yes | 1 | 3 | 0.48 |
| **No** | 5 | 5 |  |
| Cannot answer without more information | 5 | 3 |  |
| What are the consequences of incomplete treatment? | | | |
| Development of resistant tuberculosis | 4 | 1 | 0.31 |
| Failure to fully cure the disease | 0 | 1 |  |
| Further transmission of the disease | 0 | 0 |  |
| **All of the above** | 7 | 9 |  |

*the correct response is bolded

| Table 4. Responses to TB KAP survey attitude questions pre- and post-training* (n=11) | | | |
| --- | --- | --- | --- |
| **Question** | **Pre-training**  **(No. who selected response)** | **Post-training  (No. who selected response)** | **Wilcoxon rank-sum test p-value** |
| ***Attitudes & beliefs*** | | | |
| Finding every new case of tuberculosis is essential for control of the disease | | | |
| Strongly agree | 6 | 6 | 0.82 |
| Agree | 4 | 5 |  |
| Neutral | 1 | 0 |  |
| Disagree | 0 | 0 |  |
| Strongly Disagree | 0 | 0 |  |
| Community engagement is essential for the control of the disease | | | |
| Strongly agree | 8 | 10 | 0.34 |
| Agree | 2 | 1 |  |
| Neutral | 1 | 0 |  |
| Disagree | 0 | 0 |  |
| Strongly Disagree | 0 | 0 |  |
| There is a substantial increase in treatment completion rates if direct observed treatment is used | | | |
| Strongly agree | 5 | 6 | 1.0 |
| Agree | 4 | 2 |  |
| Neutral | 2 | 3 |  |
| Disagree | 0 | 0 |  |
| Strongly Disagree | 0 | 0 |  |
| There is a stigma associated with tuberculosis in Kwazulu-Natal | | | |
| Strongly agree | 3 | 5 | 0.33 |
| Agree | 6 | 5 |  |
| Neutral | 0 | 1 |  |
| Disagree | 1 | 0 |  |
| Strongly Disagree | 1 | 0 |  |
| The way you interact with tuberculosis patients can contribute to any existing stigma | | | |
| Strongly agree | 3 | 5 | 0.72 |
| Agree | 5 | 4 |  |
| Neutral | 1 | 0 |  |
| Disagree | 2 | 2 |  |
| Strongly Disagree | 0 | 0 |  |
| Tuberculosis as a disease has more stigma associated with it than HIV | | | |
| Strongly agree | 2 | 3 | 0.44 |
| Agree | 2 | 2 |  |
| Neutral | 2 | 2 |  |
| Disagree | 3 | 4 |  |
| Strongly Disagree | 2 | 0 |  |
| Directly observed treatment (DOT) is more effective than education in ensuring patient adherence to TB treatment | | | |
| Strongly agree | 6 | 5 | 0.89 |
| Agree | 2 | 2 |  |
| Neutral | 1 | 2 |  |
| Disagree | 2 | 2 |  |
| Strongly Disagree | 0 | 0 |  |
| In Kwazulu-Natal, the general population is aware of the tuberculosis services that are available | | | |
| Strongly agree | 1 | 0 | 0.47 |
| Agree | 3 | 3 |  |
| Neutral | 4 | 3 |  |
| Disagree | 3 | 5 |  |
| Strongly Disagree | 0 | 0 |  |
| Public awareness regarding tuberculosis as a health problem in Kwazulu-Natal is adequate | | | |
| Strongly agree | 2 | 0 | 0.14 |
| Agree | 3 | 2 |  |
| Neutral | 0 | 1 |  |
| Disagree | 6 | 7 |  |
| Strongly Disagree | 0 | 1 |  |
| Multi-drug resistant tuberculosis is a problem in Kwazulu-Natal | | | |
| Strongly agree | 1 | 4 | 0.28 |
| Agree | 5 | 4 |  |
| Neutral | 5 | 3 |  |
| Disagree | 0 | 0 |  |
| Strongly Disagree | 0 | 0 |  |
| Traditional or alternative medicine assists in wellbeing of tuberculosis patients | | | |
| Strongly agree | 2 | 0 | 0.02 |
| Agree | 1 | 0 |  |
| Neutral | 3 | 1 |  |
| Disagree | 4 | 3 |  |
| Strongly Disagree | 1 | 7 |  |
| Standard first-line treatment for drug-sensitive tuberculosis is accepted by patients | | | |
| Strongly agree | 2 | 1 | 1.0 |
| Agree | 5 | 6 |  |
| Neutral | 3 | 4 |  |
| Disagree | 1 | 0 |  |
| Strongly Disagree | 0 | 0 |  |
| In Kwazulu-Natal, there are many barriers to tuberculosis treatment | | | |
| Strongly agree | 0 | 3 | 0.01 |
| Agree | 4 | 6 |  |
| Neutral | 6 | 1 |  |
| Disagree | 1 | 1 |  |
| Strongly Disagree | 0 | 0 |  |
| Making people with possible/confirmed pulmonary tuberculosis wear masks in the clinic is acceptable | | | |
| Strongly agree | 6 | 3 | 0.66 |
| Agree | 2 | 6 |  |
| Neutral | 2 | 1 |  |
| Disagree | 1 | 1 |  |
| Strongly Disagree | 0 | 0 |  |
| Teaching tuberculosis patients cough hygiene is not important | | | |
| Strongly agree | 0 | 1 | 1.0 |
| Agree | 0 | 0 |  |
| Neutral | 0 | 0 |  |
| Disagree | 3 | 0 |  |
| Strongly Disagree | 8 | 10 |  |
| Infection control is an important means to prevent contracting tuberculosis | | | |
| Strongly agree | 7 | 6 | 0.77 |
| Agree | 4 | 5 |  |
| Neutral | 0 | 0 |  |
| Disagree | 0 | 0 |  |
| Strongly Disagree | 0 | 0 |  |
| I have been seriously concerned I have had tuberculosis | | | |
| Strongly agree | 1 | 2 | 0.30 |
| Agree | 4 | 4 |  |
| Neutral | 0 | 1 |  |
| Disagree | 2 | 2 |  |
| Strongly Disagree | 4 | 2 |  |
| I should know whether I’ve got or had tuberculosis | | | |
| Strongly agree | 4 | 5 | 0.41 |
| Agree | 5 | 6 |  |
| Neutral | 2 | 0 |  |
| Disagree | 0 | 0 |  |
| Strongly Disagree | 0 | 0 |  |
| If I contracted tuberculosis, I would be allowed to continue working in my current capacity | | | |
| Strongly agree | 2 | 1 | 0.93 |
| Agree | 6 | 7 |  |
| Neutral | 1 | 1 |  |
| Disagree | 2 | 2 |  |
| Strongly Disagree | 0 | 0 |  |
| My employer would maintain confidentiality if I were to contract tuberculosis | | | |
| Strongly agree | 3 | 3 | 0.93 |
| Agree | 3 | 5 |  |
| Neutral | 3 | 1 |  |
| Disagree | 1 | 1 |  |
| Strongly Disagree | 1 | 1 |  |
| I should know my own HIV status | | | |
| Strongly agree | 11 | 10 | 1.0 |
| Agree | 0 | 1 |  |
| Neutral | 0 | 0 |  |
| Disagree | 0 | 0 |  |
| Strongly Disagree | 0 | 0 |  |

| Table 5. Treatment initiation after bacteriologically confirmed TB diagnosis during study and historical control periods* | | |
| --- | --- | --- |
| **Period** | **Intervention  (May 21 to Sep 4, 2018)** | **Historical control (May 22 to Sep 5, 2017)** |
| Total bacteriologically confirmed DS-TB cases (%) | 102 (100) | 84 (100) |
| Started TB treatment (%)** | 94 (92.2) | 68 (81.0) |
| **Unadjusted difference in proportion initiating treatment (95% CI):** 11.2 (0.2-22.2) **Adjusted difference in proportion initiating treatment (95% CI):** 10.1 (1.5 to 21.3)*** | | |
| Median treatment delay**** (IQR) | 4.5 (3) | 7 (4.3) |
| **Unadjusted difference in median treatment delay (95%CI):** 2.0 (1.0 to 4.0)***** | | |

*This excludes clinically confirmed TB cases (e.g. diagnosed via x-ray or symptoms and contact history).
** Patients who had not initiated treatment by September 28, 2018 were considered as having not started TB treatment. 4/94 (4.3%) and 3/69 (4.3%) started treatment as an inpatient at a tertiary hospital during the intervention and historical control period, respectively.
***Adjusted for age, sex and calendar month using a multivariate binomial regression model with an identity link.
****The number of days between testing and starting treatment among bacteriologically confirmed TB patients who were tested during the study period and initiated treatment by September 28, 2018.
*****Estimated using a Mann-Whitney non-parametric test.

| Table 6. Details on counselling sessions | | | |
| --- | --- | --- | --- |
| **Among enrolled patients (n=84)** | | | |
|  | **Counselling session** | |  |
|  | **1** | **2** |  |
| **No. completed (%)** | 84 (100) | 58 (69.0) |  |
| **Duration (mins)** |  |  |  |
| Mean (SD) | 19.6 (6.7) | 19.1 (7.4) |  |
| Missing (%) | 9 (10.7) | 4 (6.6) |  |
| **Time since start of treatment (days)** |  |  |  |
| Mean (SD) | 5.1 (11.8) | 44.7 (25.0) |  |
| Median (range) | 0 (0 - 59) | 42 (7-147) |  |

| Table 7. Patient responses to Yes/No questions on the exit survey (n=57) | | |
| --- | --- | --- |
| **Question** | **No. of respondents** | **Positive responses (%)** |
| Found the sessions helpful (%) | 57 | 57 (100) |
| Liked the counsellor (%) | 55 | 55 (100) |
| Found them too long (%) | 54 | 9 (16.7) |
| Found them to be convenient (%) | 52 | 47 (90.4) |
| Felt comfortable asking questions (%) | 54 | 53 (98.1) |
| Felt comfortable sharing problems (%) | 53 | 52 (98.1) |
| The sessions increased confidence to talk to family about TB (%) | 53 | 53 (100) |
| Happy with the counsellors' answers to questions (%) | 53 | 53 (100) |
| Confident in finishing treatment (%) | 53 | 53 (100) |
| The sessions increased confidence (%) | 50 | 50 (100) |
| Preferred the 1^st^ session to the 2^nd^ session (%) | 41 | 27 (65.9) |

| Table 8. Comparing the proportion of patients who successfully completed TB treatment | | |
| --- | --- | --- |
| **Comparison** | **Unadjusted risk difference (95%CI)** | **Adjusted risk difference (95%CI)*** |
| *Intention-to-treat analysis:*  Intervention vs historical control period | 0.6% (-11.5 to 12.5%) | 4.4% (-7.3 to 16.0%) |
| *Per protocol analysis:*  Enrolled in study vs all others in historical and control period | 11.2% (-1.6 to 23.8%) | 11.6% (-0.8 to 24.0%) |
| *Modified per protocol analysis:* Enrolled in study vs historical control period | 8.1% (-6.1 to 22.1%) | 7.9% (-5.9 to 21.7%) |
| *Intervention period only*:  Enrolled vs unenrolled in study | 15.6% (0.5 to 30.7%) | 13.9 (-0.2 to 28.1%) |

*Adjusted for baseline patient characteristics: age, sex, HIV and ART status, smear status
